# Irisin suppresses pancreatic β cell pyroptosis in T2DM by inhibiting the NLRP3-GSDMD pathway and activating the Nrf2-TrX/TXNIP signaling axis

**DOI:** 10.1186/s13098-023-01216-5

**Published:** 2023-11-22

**Authors:** Tianrong Li, Jingjing Yang, Anjun Tan, Hewen Chen

**Affiliations:** Department of Geriatric Medicine, The First People’s Hospital of Yunnan Province, The Affiliated Hospital of Kunming University of Science and Technology, No. 157 Jinbi Road, Kunming, Yunnan, 650032 China

**Keywords:** Irisin, T2DM, Pyroptosis, NLRP3, GSDMD, Nrf2-TrX/TXNIP

## Abstract

**Background:**

Irisin plays a key role in metabolic diseases, including type 2 diabetes mellitus (T2DM). However, the mechanism underlying the link between irisin and the development of T2DM, particularly in pancreatic islet β-cells, remains unknown.

**Methods:**

In vitro, Min6 cells were treated with high glucose (HG) to generate T2DM cell models. GSDMD-N staining, Western blotting assays, and ELISA were performed to measure the expression levels of GSDMD, caspase 1, IL-1β, and IL-18. Next, the NLRP3 stimulator, ATP, was used to assess the effect of irisin on NLRP3 inflammasome. To evaluate the function of the Nrf2-TrX/TXNIP signaling axis, the Nrf2 inhibitor ML385 was used. For in vivo assessment, we first established T2DM model mice. Then, hematoxylin and eosin (H&E) staining was performed to observe the islet morphology, and the immunofluorescence technique was used to examine the mass of α and β cells. To confirm the role of the Nrf2-TrX/TXNIP signaling axis, ML385 was injected into the mice. Immunofluorescence of Nrf2, caspase 1, and GSDMD was detected in the islet cells of the model mice to verify the results.

**Results:**

We found that irisin treatment significantly decreased the expression of GSDMD-N (P31) and cleaved caspase-1 (p20), decreased caspase1 activity, and inhibited the secretion of IL-1β and IL-18 in HG-treated Min6 cells. We also found that irisin inhibited oxidative stress and NLRP3 expression by activating the Nrf2-TrX/TXNIP signaling axis. Additionally, in the T2DM model mice, irisin enhanced the function of islet cells, decreased insulin resistance, and preserved the morphology of pancreatic islets.

**Conclusion:**

We showed in this study that irisin can be used for treating pyroptosis in HG-induced islet β-cells and T2DM model mice. We also found that irisin inhibits pyroptosis and oxidative stress by inhibiting the NLRP3-GSDMD pathway and activating the Nrf2-TrX/TXNIP signaling axis.

**Supplementary Information:**

The online version contains supplementary material available at 10.1186/s13098-023-01216-5.

## Background

Diabetes mellitus is a serious global health concern. It affects nearly 536.6 million adults worldwide. Specifically, type 2 diabetes mellitus (T2DM), the most prevalent form of diabetes [1], is associated with a substantially greater risk of all-cause mortality [2–5]. Thus, further research on the mechanisms and risk factors for T2DM is required for developing effective prevention and treatment strategies.

T2DM is characterized by a functional deficit of insulin, resulting from an imbalance between insulin levels and insulin sensitivity [6]. Insulin resistance is a multifactorial condition commonly attributed to obesity and aging [7]. Irisin is a type of polytropic myokine, with a polypeptide chain containing 112 amino acids. Irisin is released by the skeletal muscles of mice and humans during exercise. It primarily targets adipose tissue and induces a process referred to as the ‘Browning’ of white adipose tissue [8, 9]. Following its discovery in 2012, irisin has been found to be closely associated with various metabolic diseases, including T2DM [10, 11]. In most observational studies, irisin expression decreases in patients with T2DM [12]. Most studies provide evidence supporting a positive correlation between irisin and the obesity index [13]. A moderate increase in the circulating irisin levels, can effectively enhance energy expenditure, prevent weight gain caused by dietary factors, and reduce insulin resistance [14]. Irisin can mitigate the insulin resistance of lipotoxic-induced mouse β-cells via the PI3K/AKT/FOXO1 axis [15]. In mouse experiments, irisin has also been shown to activate integrin-αv/β5 and ERK 1/2 to reduce T2DM-related complications [16, 17]. These findings suggest that irisin is a promising target for treating T2DM, although further investigation is still needed.

The pancreatic β-cells are responsible for secreting insulin. They coordinate with glucagon-secreting α-cells to control blood glucose levels. The progression of T2DM is typically accompanied by β-cell dysfunction [18]. This occurs mainly due to the loss of β-cells and the dysfunction of several molecular pathways. Pyroptosis, a form of inflammatory cell death mediated by inflammasome, plays a key role in T2DM. The NLRP3 inflammasome consists of apoptosis-associated speck-like proteins, such as caspase recruitment domain (ASC) and caspase-1. Several researchers have extensively studied its role as a key promoter in this process [19]. Inflammatory caspase cleaves the C-terminal and N-terminal regions of GSDMD, following which, the N-terminal domain oligomerizes on the cell membrane to form membrane pores. This leads to the release of IL-18 and IL-1β, which promote β-cell apoptosis and decrease insulin secretion [20]. However, the mechanism underlying the regulatory function of the inflammasome in T2DM needs further investigation. Besides inflammation, high oxidative stress is also a significant factor that contributes to a decrease in glucose tolerance and the activation of the NLRP3 inflammasome observed in patients with T2DM [21]. Nuclear factor erythroid-derived 2-like 2 (Nrf2) is a significant target in disease management as it plays a key role in regulating antioxidant responses. Some studies have shown that Nrf2 can enhance neuroprotection and insulin sensitivity in both its upstream and downstream targets [22]. Also, the Nrf2/thioredoxin (Trx) axis represents a specific therapeutic target for the treatment of injury or cell death. Thioredoxin-interacting protein (TXNIP) is the most potent glucose-responsive gene in pancreatic β-cells. It is located at position 1q21–1q23 on the chromosome within the T2DM locus. TXNIP plays a key role in activating the NLRP3 inflammasome, thus, assessing its function may provide valuable insights into the association between IL-β and the development of T2DM [19].

As Irisin plays an important role in improving blood glucose and insulin resistance, we hypothesized that it may protect the islet cells by affecting oxidative stress and the NLRP3-mediated pyroptosis pathway via the Nrf2-Trx/TXNIP axis. In this study, we elucidated the mechanism of action of irisin and provided fundamental data for the clinical translational treatment of human metabolic diseases and other diseases that can be ameliorated through exercise.

## Methods

### Materials/Chemicals

The cell culture medium RPMI-1640, fetal bovine serum (FBS), penicillin-streptomycin solution (dual antibody; BL505A), and β-mercaptoethanol (M301574) were purchased from VivaCell (Shanghai, China), Biosharp (Hefei, China), and Aladdin (Shanghai, China). D-(+)-Glucose (≥ 99.5%), and ATP (D7378) were purchased from Beyotime (Shanghai, China). Irisin (HY-P70664) and ML385 (HY-100,523) were purchased from MCE (NJ, USA). Streptozotocin (STD) was purchased from Solarbio (Beijing, China). The DCFH-DA Reactive oxygen ROS fluorescent probe was purchased from Meilunbio® (Dalian, China). Carboxyl -H2DCFDA (C400) and Carboxyl -DCFDA were purchased from Invitrogen (USA).

ELISA kits for insulin (INS) (CSB-E05071 m), interleukin 18 (IL-18; CSB-E04609 m), and IL-1β (CSB-E08054 m) were purchased from CUSABIO (Wuhan, China). The Caspase 1 Activity Assay Kit (C1101), Glucagon ELISA Kit, and hematoxylin-eosin (HE) staining kit were purchased from Beyotime. The kits for detecting malondialdehyde (MDA; A003-4-1) and superoxide dismutase (SOD; A001-3-2) were purchased from Nanjing Jiancheng Bioengineering Research Institute (China). PrimeScript™ RT Kit was purchased from Qiagen (Germany).

Anti-GSDMD N-Terminal (DF13758), anti-GSDMD (20770-1-AP), anti-Nrf2 (GB113808), anti-FNDC5 (23995-1-AP), anti-β-actin (AB0035), anti-NLRP3 (DF7438), anti-ASC (DF6304), anti-Lamin B1 (12232-1-AP), anti-TXNIP (18243-1-AP), anti-TrX1 (AF7577), anti-DAPI (G1012), anti-Insulin (GB13121), anti-glucagon (GB11097), anti-caspase1 (af5418), anti-Cleaved-Caspase 1 (Asp296), and p20 (AF4005) were purchased from Proteintech (Wuhan, China), Abways (Beijing, China), Servicebio (Wuhan, China), and Affinity Biosciences (Jiangsu, China).

### T2DM pancreatic β-cell model

The mouse islet β-cells Min6 (FH0390) were purchased from FuHeng (Shanghai, China). The complete growth medium for Min6 consisted of 89% RPMI-1640, 10% FBS, 1% dual antibody, and 0.05 mM β-mercaptoethanol. To induce the T2DM cell model, the Min6 cells were exposed to a high glucose (HG; 25 mmol/L) condition for 24 h. Then, ELISA was performed to evaluate the effectiveness of the modeling process.

### Cell group

To investigate the effect of irisin, Min6 cells were divided into six groups, including the Control group, HG group, HG + PBS group, HG + irisin group, HG + irisin + ATP group, and HG + irisin + ML385 group. The cells in the Control group were cultured in a normal medium supplemented with PBS, the cells in the HG group were cultured in the HG medium supplemented with PBS, and the cells in the HG + irisin group were cultured in the HG medium supplemented with 100 ng/mL irisin. The processing time was 24 h. The cells in the ATP treatment group were additionally treated with 3 mM ATP for 45 min. The cells in the ML385 treatment group were treated with 2 μM ML385 for 30 min.

### T2DM mouse model and collection of biological indicators

Animal experiments were conducted following protocols approved by the First People’s Hospital of Yunnan Province, the Affiliated Hospital of Kunming University of Science and Technology. Male C57BL/6J mice were purchased from Bei You Biotechnology. All mice were raised in metabolic cages at 22 °C, and a 12-h/12-h light-dark cycle was maintained.

The mice were divided into five groups at random (N = 6 mice/group): the Control group, Model group, Model + PBS group, Model + Irisin group, and Model + Irisin + ML385 group. A high-fat diet (HFD, 45% kcal, MS1607, Shanghai Maokang Biotechnology Co., Ltd., China) plus low-dose STD was used to construct the T2DM mouse model. When the mice were five weeks old, they were fed HFD consisting of 45 kcal% for an additional four weeks. The mice induced with insulin resistance were given a single intraperitoneal injection of 50 mg/kg STZ. The fasting blood glucose level (FBG) equal to or greater than 11.1 mmol/L was classified as diabetic [23]. The diabetic mice in the Model + Irisin group were administered a peritoneal injection of irisin (0.25 μg/kg/day) for 21 days. The diabetic mice in the Model + Irisin + ML385 group were administered a peritoneal injection of irisin (0.25 μg/kg/day) along with ML385 (30 mg/kg/day) for 21 days. In the control group, mice were given a basal diet and administered an intraperitoneal injection of an equal amount of 0.1 mmol/L sodium citrate buffer. For ML385 treatment, mice were intraperitoneally injected with ML385 (30 mg/kg/day) for three weeks. Blood samples were collected from the tail vein of the mice for further analysis.

The oral glucose tolerance test (OGTT) of the mice was performed at the end of the intervention. Briefly, after the mice were starved for 15 h and fed glucose via oral gavage (2 g/kg), their fasting plasma insulin (FINS) and FBG (at 0, 30, 60, and 120 min) levels were measured using ELISA kits. The levels were evaluated using the following equations: HOMA-β = 20×FINS/(FBG-3.5), HOMA-IR = FBG× FINS/22.5.

The mice were euthanized by carbon dioxide (CO_2_) asphyxiation. Their pancreas was isolated by collagenase digestion of the pancreas and snap-frozen at − 80 °C for subsequent assays. Some of the pancreatic tissues were fixed in 4% paraformaldehyde and subsequently processed to form paraffin samples. These samples were pre-cut to a thickness of 6 μm using a slicer (RM2016, Leica, Germany).

### Western blotting analysis

The Min6 cells were washed with PBS to remove the culture medium, and then, they were lysed with a RIPA lysis buffer on ice for 8 min. The pancreatic tissues were initially snipped and then subjected to lytic ultrasound treatment. The protein concentration was assessed by adding BCA solution. The protein samples, which were denatured in advance, were electrophoretically separated on polyacrylamide gel. Following this, the proteins were electrically transferred onto a nitrocellulose membrane. The membranes were washed and blocked at room temperature for 1 h using non-fat powdered milk. They were incubated with primary antibodies overnight at 4 °C. The following day, they were incubated with secondary antibodies at room temperature for 1 h. Finally, color reaction was performed using the pre-mixed ECL luminescent substrate.

### Caspase 1 activity

The cells were collected after incubation for 48 h. The enzyme activity of caspase 1 was measured using a Caspase 1 Activity Assay Kit following the manufacturer’s instructions. Briefly, the cells were mixed with lysate on ice to be cleaved for 15 min and then incubated with Ac-YVAD-pNA (2 mM) at 37 °C for 80 min. The OD value was recorded at 405 nm using a spectrophotometer (1510, Thermo Fisher).

### ELISA

The cell supernatants were collected after incubation for 48 h. The level of insulin and the secretion of IL-1β and IL-18 in the cell supernatants or mouse serum samples were measured using an ELISA kit following the manufacturer’s instructions. Briefly, the diluted cell supernatant was introduced into the bottom of the well coated with monoclonal antibodies and left to incubate at 37 °C for 1 h. Subsequently, the enzyme-labeled solution was incubated for another 1 h. The color reaction was conducted for 10 min, and it was terminated using a stop solution. The OD value was recorded at 450 nm using a spectrophotometer (1510, Thermo Fisher).

### Oxidative stress assays

The cells were collected and incubated with 5 μM DCFH-DA probe, which was diluted in serum-free medium, in a wet box for 30 min at 37 °C. The images were captured by the THUNDER Imager (Leica, Germany).

For pancreatic tissues, 100 μg of total protein was incubated at 37 °C with either oxidation-sensitive carboxy-H2DCFDA (C400) or oxidation-insensitive carboxy-DCFDA (C369); the final concentration of both was 25 μM. The measurements were recorded at 0 and 30 min using a fluorescent enzyme-labeled apparatus with excitation at 485 nm and emission at 530 nm. The data were expressed as multiples of the C400/C369 ratio. The cell lysate or mouse serum samples were used to determine the MDA and SOD levels using specific reagent kits, and the OD values were read at 530 nm and 450 nm, respectively.

### Immunofluorescence staining

The sections of cells and pancreatic tissues first underwent antigen repair with EDTA (pH 9.0) for 27 min, and then, they were sealed in goat serum for 30 min. The sections were blocked and added with diluted antibodies, and subsequently, incubated in a wet box at 4 °C overnight. The following day, the corresponding fluorescently labeled secondary antibodies were added respectively and incubated for another 50 min in the wet box. DAPI was used to re-stain the nucleus. The fluorescence intensity of GSDMD (Green) was measured to assess the level of pyroptosis. The fluorescence intensity of insulin and glucagon was measured to assess pancreatic function. The distribution of insulin (red) and glucagon (green) was observed by fluorescence microscopy. The β-cells are the source of insulin, and α cells are the source of glucagon. Immunohistochemical double-labeling of caspase-1 and islet β-cells was performed.

### Histological staining

The sections of the pancreas were stained using the H&E staining kit. Briefly, the samples were incubated with hematoxylin for 4 min, followed by the application of eosin solution for 30 s. The images were captured using an inverted microscope (CKX3-SLP, Olympus).

### Statistical analysis

The data were analyzed using GraphPad Prism version 8.0. All experiments were performed with at least three replicates. The outcomes were presented as the mean ± SD, and the statistical differences were assessed by Student’s* t*-test or ANOVA. Image J was used for the semi-quantitative analysis. All results were considered to be statistically significant at *P* < 0.05.

## Results

### Irisin inhibited pyroptosis of Min6 cells treated with HG

**Fig. 1:**
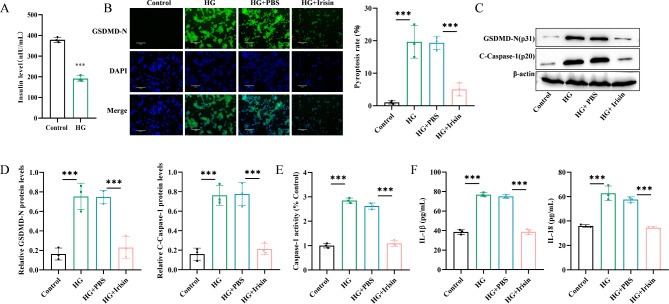
Irisin inhibited cellular pyroptosis in Min6 cells treated with high glucose. Min6 cells (mouse pancreatic islet β-cells) were incubated with 25 mmol/L glucose for 24 h to construct a T2DM cell model. (**A**) ELISA was performed to determine the insulin level to evaluate whether the modeling was successful. (**B**) GSDMD-N immunofluorescence staining was performed to detect cellular pyroptosis after adding 100 ng/mL irisin, followed by incubation for 24 h. (**C**) Western blotting assays were performed to detect the levels of C-caspase-1 and GSDMD-N. (**D**) Semi-quantitative analysis of proteins was performed. (**E**) Caspase-1 activity was detected. (**F**) ELISA was performed for detecting the level of secretion of IL-1β and IL-18 proteins; ***p < 0.001

A T2DM cell model was established by incubating Min6 cells with 25 mmol/L glucose for 24 h. Whether the development of the model was successful was evaluated by measuring the level of insulin in the culture medium using ELISA (Fig. 1A). Based on our previous study, the optimal concentration of irisin for Min6 cells was determined to be 100 ng/mL [24]. This concentration of irisin was most effective in promoting glucose-stimulated insulin secretion in Min6 cells. The immunofluorescence images indicated a considerable increase in the level of expression of GSDMD-N (P31) in HG-treated Min6 cells, whereas the images of the cells in the HG + Irisin group suggested a significant reduction in the level of expression of GSDMD-N (P31) (Fig. 1B). Additionally, the level of expression of the GSDMD-N (P31) and cleaved caspase-1 (p20) (C-caspase-1) proteins increased significantly following HG treatment, but the level of both proteins decreased significantly after treatment with irisin (Fig. 1C and D). Caspase1 activity in Min6 cells increased significantly after HG treatment, as observed using the Caspase1 Activity Assay Kit. However, the addition of irisin significantly decreased caspase1 activity (Fig. 1E). The secretion levels of IL-1β and IL-18 also increased in HG-treated Min6 cells, but their levels decreased significantly in the HG + Irisin group (Fig. 1F).

### Irisin inhibited oxidative stress and pyroptosis of Min6 cells treated with HG by downregulating the expression of NLRP3

**Fig. 2:**
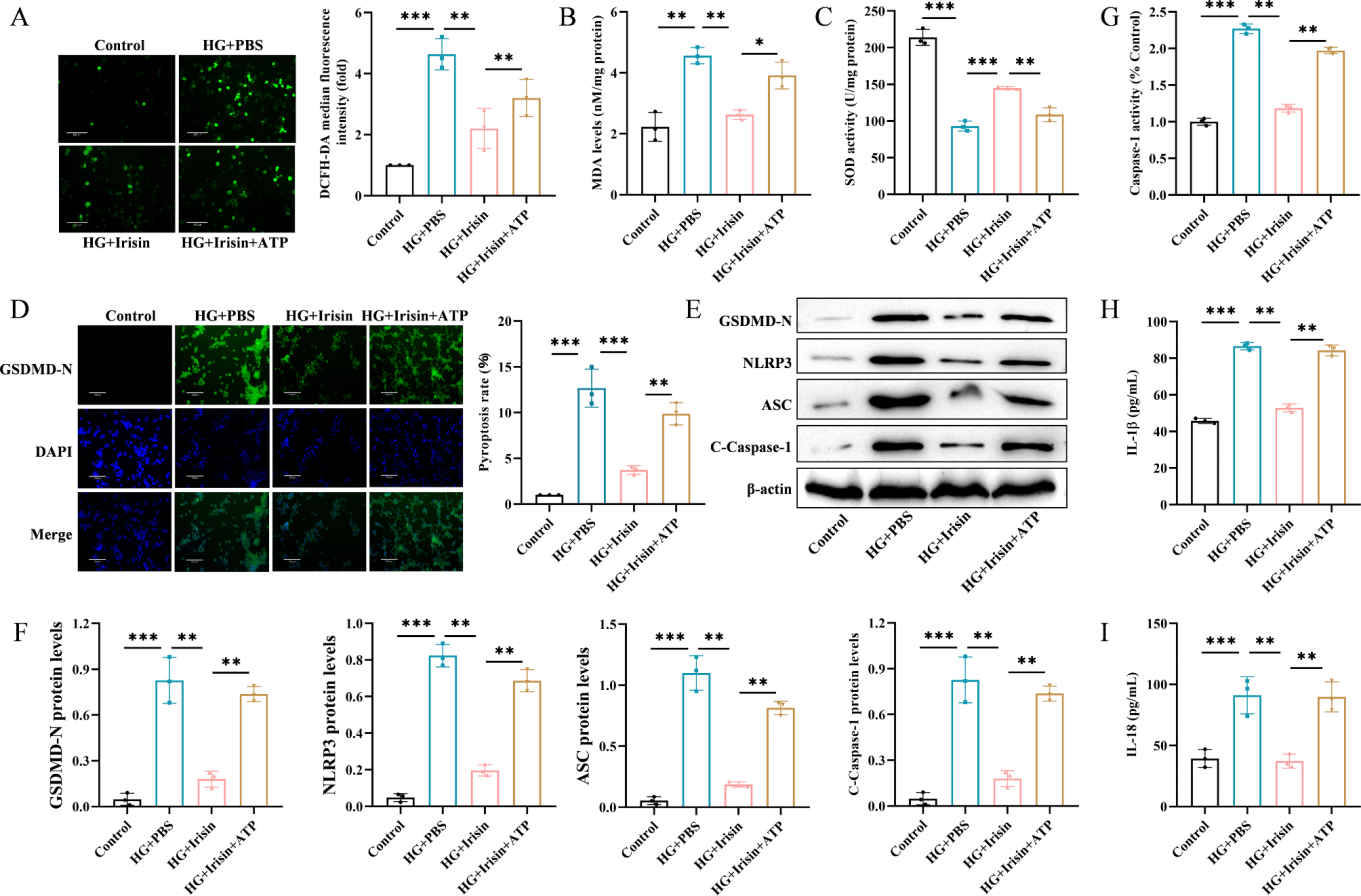
Irisin inhibited oxidative stress and pyroptosis in HG-treated Min6 cells by downregulating the expression of NLRP3. Min6 cells were incubated with 25 mmol/L glucose for 24 h to construct a T2DM cell model. The medium was changed to that contained irisin (100 ng/mL), and incubation was continued for 24 h. Next, 3 mM ATP was added to the medium and incubated for 45 min to activate the expression of NLRP3, and then, indices reflecting oxidative stress (**A**: ROS, **B**: MDA, and **C**: SOD) were assessed. (**D**) GSDMD-N immunofluorescence staining was performed to detect cellular pyroptosis; (**E**) Western immunoblotting assay was performed to evaluate the level of expression of GSDMD-N, NLRP3, ASC, and C-caspase-1 proteins. (**F**) Protein quantitative analysis was performed. (**G**) Caspase-1 activity was determined using the Caspase 1 Activity Assay Kit ; ELISA was performed to determine the level of expression of IL-1β (**H**) and IL-18 (**I**) to evaluate the inhibitory effect of irisin on HG-induced oxidative stress and pyroptosis in Min6 cells. Scale bar = 130 μm; **p < 0.01 and ***p < 0.001

We investigated the mechanism by which irisin inhibited oxidative stress and cellular pyroptosis in HG-treated Min6 cells mediated by the suppression of NLRP3 inflammasome. We used ATP, an agonist of NLRP3, to activate the expression of NLRP3. We first examined reactive oxygen species (ROS) production, malondialdehyde (MDA) levels, and superoxide dismutase (SOD) activity. We found that HG treatment significantly increased the production of oxidized ROS and MDA but decreased the SOD activity. However, Irisin significantly inhibited the release of ROS, decreased MDA levels, and enhanced SOD activity. After ATP treatment, the effect of irisin was significantly reversed (Fig. 2A-C). The results of the immunofluorescence experiments showed that ATP significantly increased the expression of GSDMD-N. The results of the Western blotting assay showed that the expression levels of GSDMD-N, NLRP3, ASC, and cleaved caspase-1 were significantly increased after HG treatment, but these effects were reversed by irisin. Irisin-mediated changes in the expression of GSDMD-N, NLRP3, ASC, and cleaved caspase-1 proteins increased significantly after ATP treatment (Fig. 2E-F). ATP also enhanced caspase-1 activity (Fig. 2G) and stimulated the secretion of IL-1β and IL-18 (Fig. 2H and I). These results suggested that inhibiting the expression of NLRP3 induced the anti-pyroptosis effect of irisin.

### Irisin inhibited oxidative stress and NLRP3 expression by activating the Nrf2-TrX/TXNIP signal axis in HG-treated Min6 cells

**Fig. 3:**
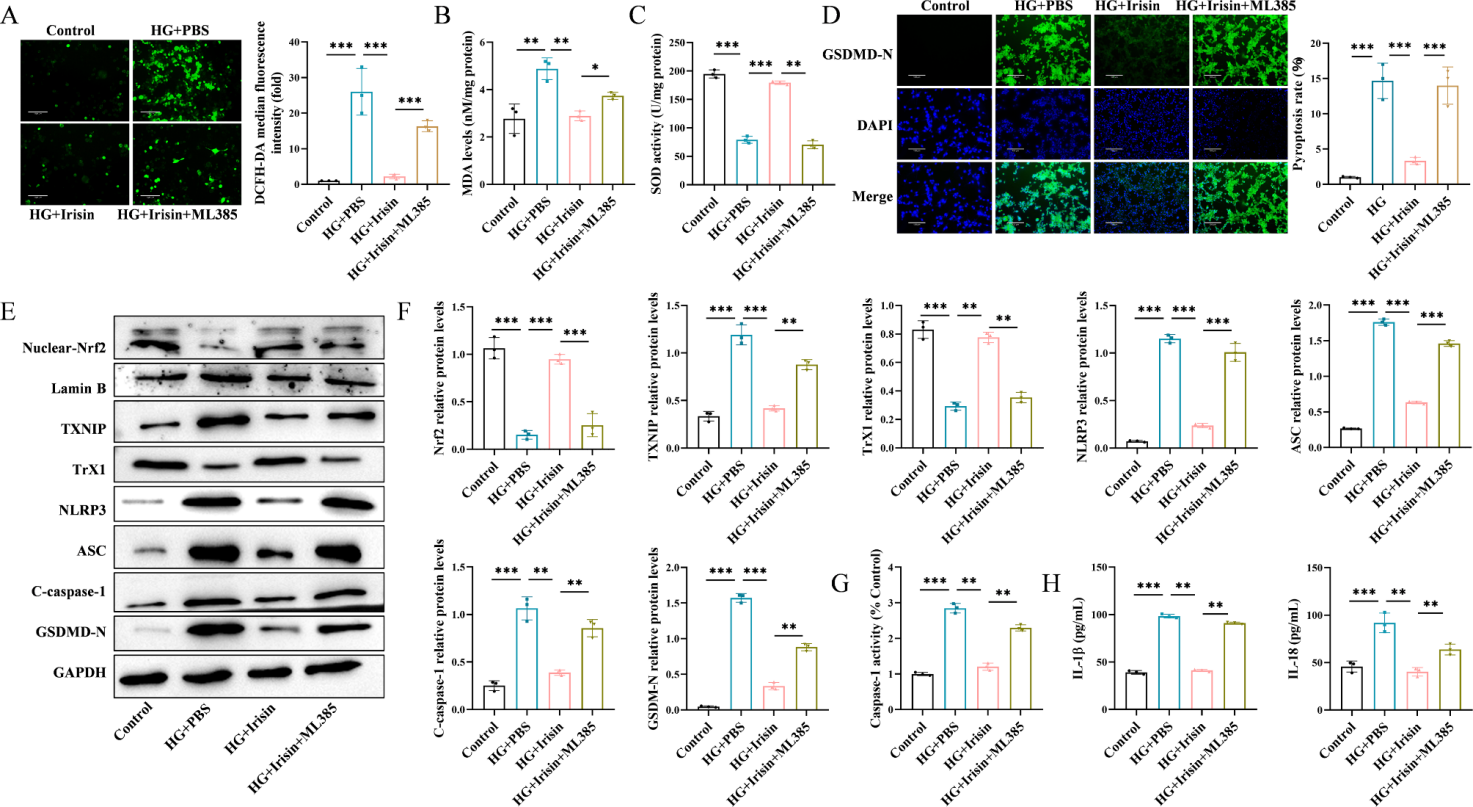
Irisin decreased oxidative stress levels and pyroptosis through activation of the Nrf2-TrX/TXNIP signaling axis The T2DM cell model was established via HG induction. Then, 100 ng/mL Irisin was added along with the Nrf2 inhibitor ML385, and the level of oxidative stress of cells in each group was evaluated (**A**: ROS, **B**: MDA, and **C**: SOD). (**D**) Pyroptosis was detected by GSDMD-N staining. (**E**) WB assays were performed to evaluate the level of expression of Nrf2 in the nucleus, as well as, the level of expression of TXNIP, TrX1, NLRP3, ASC, C-caspase-1, and GSDMD-N. (**F**) The protein expression levels were calculated as grayscale values. (**G**) Caspase-1 activity was assessed. ELISA was performed to assess the level of expression of IL-1β and IL-18 proteins (**H**) for elucidating the mechanism underlying the inhibition of oxidative stress and NLRP3-mediated pyroptosis by irisin. Scale bar = 130 μm; *p < 0.05, **p < 0.01, and ***p < 0.001

We investigated the mechanism by which the Nrf2-TrX/TXNIP signaling axis was involved in irisin-mediated inhibition of oxidative stress and cellular pyroptosis in HG-treated Min6 cells. We used ML385, an inhibitor of Nrf2, to suppress the expression of Nrf2. First, we examined ROS production, MDA levels, and SOD activity. We found that HG treatment significantly promoted the production of oxidized ROS and MDA but decreased SOD activity, whereas irisin significantly inhibited ROS release, decreased MDA levels, and enhanced SOD activity. After treatment with ML385, the effects of irisin were significantly reversed (Fig. 3A-C). The results of the immunofluorescence experiments showed that ML385 significantly promoted the expression of GSDMD-N that was reduced by irisin (Fig. 3D). The results of the Western blotting assays showed that the expression levels of Nrf2 in the nucleus, as well as, the expression of TrX1 decreased significantly after HG treatment, whereas, the expression of TXNIP, NLRP3, ASC, C-caspase-1, and GSDMD-N increased significantly. The expression levels of the above-mentioned proteins were reversed by irisin. Irisin-mediated changes in the expression of the TXNIP, NLRP3, ASC, C-caspase-1, and GSDMD-N proteins increased significantly again after treatment with ML385, and the expression levels of Nrf2 and TrX1 decreased significantly (Fig. 3E-F). We also found that ML385 enhanced caspase-1 activity (Fig. 3G) and promoted the secretion of IL-1β and IL-18 (Fig. 3H). These results confirmed that irisin inhibited the level of oxidative stress and cell death by activating the Nrf2-TrX/TXNIP signaling axis.

### Irisin improved islet-cell function and reduced insulin resistance in T2DM model mice

**Fig. 4:**
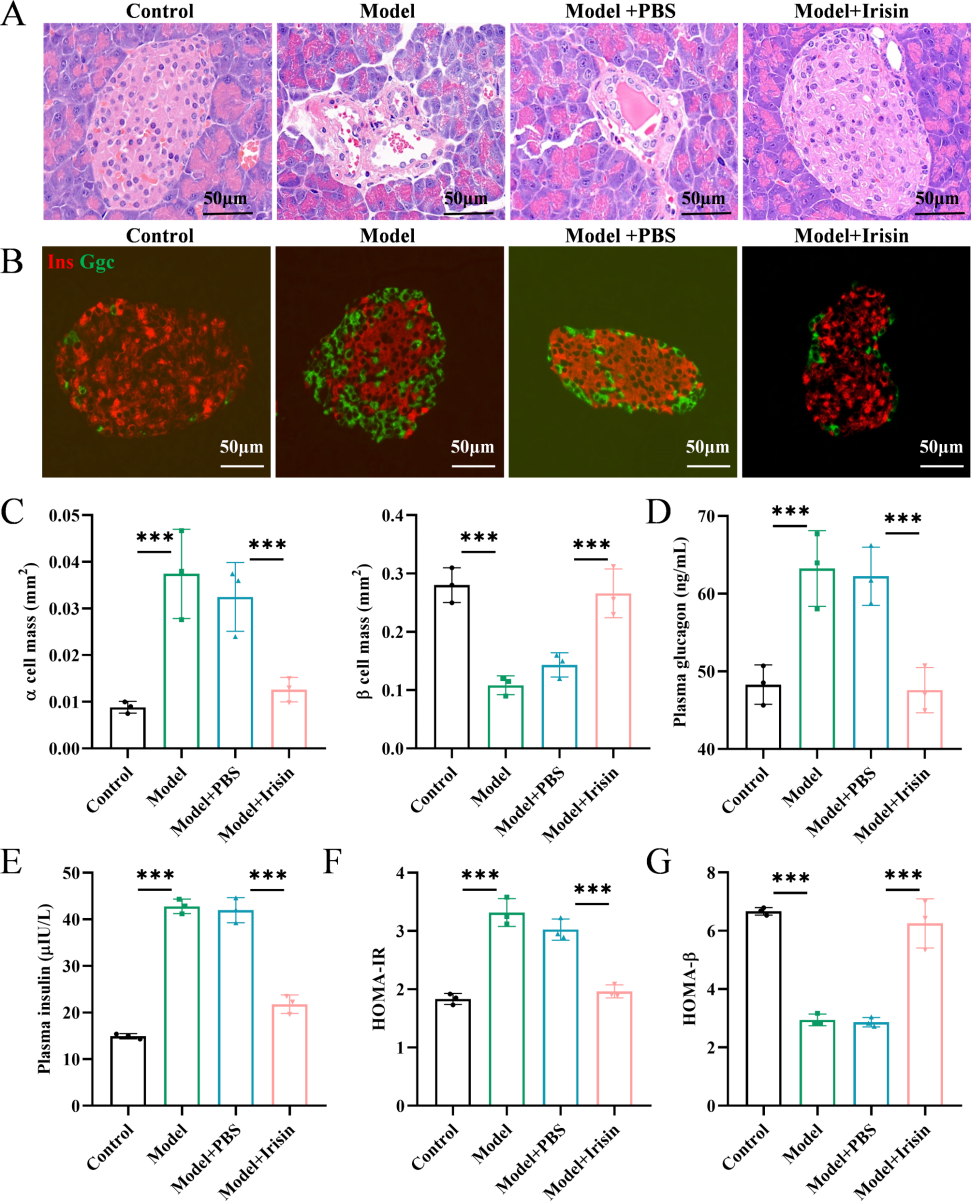
Irisin improved islet cell function and insulin resistance in T2DM model mice. (**A**) H&E staining of the mouse pancreas was performed. (**B**) Immunofluorescence detection of α and β cell mass was performed, and immunofluorescence images of islets stained with insulin (Ins) (red) and glucagon (Ggc) (green) were captured. (**C**) α and β cell mass were shown. (**D**) Fasting plasma glucagon and (**E**) insulin concentrations in mice were evaluated. (**F**) Mouse HOMA-IR and (**G**) HOMA-β values were determined. Scale bar = 50 μm; ***p < 0.001

We evaluated the effects of irisin in mice. Compared to the islets of normal mice, the islets of diabetic model mice were disorganized, entered adjacent exocrine tissues, and were infiltrated by adenoidal components, whereas, in irisin-treated mice, the islet morphology remained intact (Fig. 4A). Normal pancreatic islets of mice have a typical structure, characterized by a central β-cell aggregate and several α-cells at the periphery. In the pancreatic islets of diabetic model mice, peripheral α-cells affected the β-cell clusters, which suggested that the β-cells were converted to α-like glucagon-secreting cells. Compared to the normal mice, the model mice showed a significant increase in α-cell mass and a decrease in β-cell mass. Irisin treatment resulted in a significant decrease in α-cell mass and a significant increase in β-cell mass (Fig. 4B-C). Fasting plasma glucagon concentration increased significantly in the diabetic model mice, but it decreased significantly after irisin was added (Fig. 4D). Fasting plasma insulin concentration increased significantly in the diabetic model mice, and irisin intervention attenuated their hyperinsulinemia (Fig. 4E). Insulin resistance was assessed by evaluating the HOMA-IR values. Significant insulin resistance was recorded in the diabetic model mice; however, irisin significantly reduced insulin resistance (Fig. 4F). The β-cell function was measured by calculating HOMA-β values and was found to decrease significantly in the diabetic model mice; however, the function of these cells was largely preserved in the irisin-treated group (Fig. 4G). Thus, irisin improved islet cell function and enhanced insulin resistance in the diabetic model mice.

### Irisin inhibited oxidative stress and NLRP3 expression by activating the Nrf2-TrX/TXNIP signal axis in the T2DM model mice

**Fig. 5:**
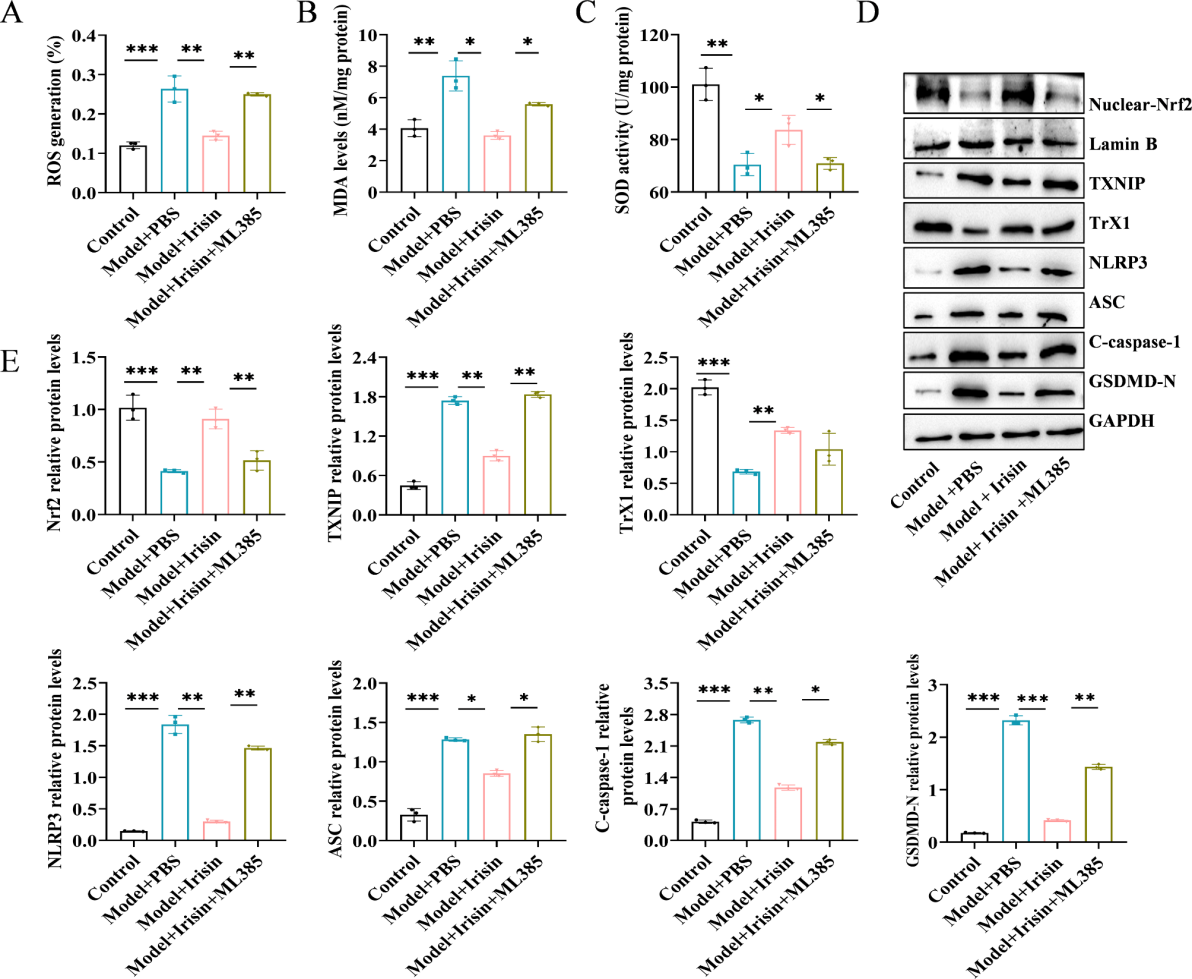
Irisin inhibited oxidative stress levels and NLRP3 expression by activating the Nrf2-TrX/TXNIP signaling axis in mice. High-fat diet + STZ-induced T2DM model mice were randomly grouped and administered irisin; the inhibitor group was injected with the Nrf2 inhibitor ML385 in vivo. (**A**-**C**) Oxidative stress levels of pancreatic tissues were detected in the mice in each group (**A**: ROS, **B**: MDA, and **C**: SOD). (**D**) WB experiments were performed to evaluate the level of expression of Nrf2, TrX, TXNIP, NLRP3, ASC, C-caspase-1, IL-1β, and GSDMD-N in pancreatic islet tissues and confirm that irisin inhibits oxidative stress and the pyroptosis of NLRP3-mediated pancreatic islet β-cells by activating the Nrf2-TrX/TXNIP signaling axis. (**D**) Protein semi-quantitative analysis was performed; **p < 0.01 and ***p < 0.001

**Fig. 6:**
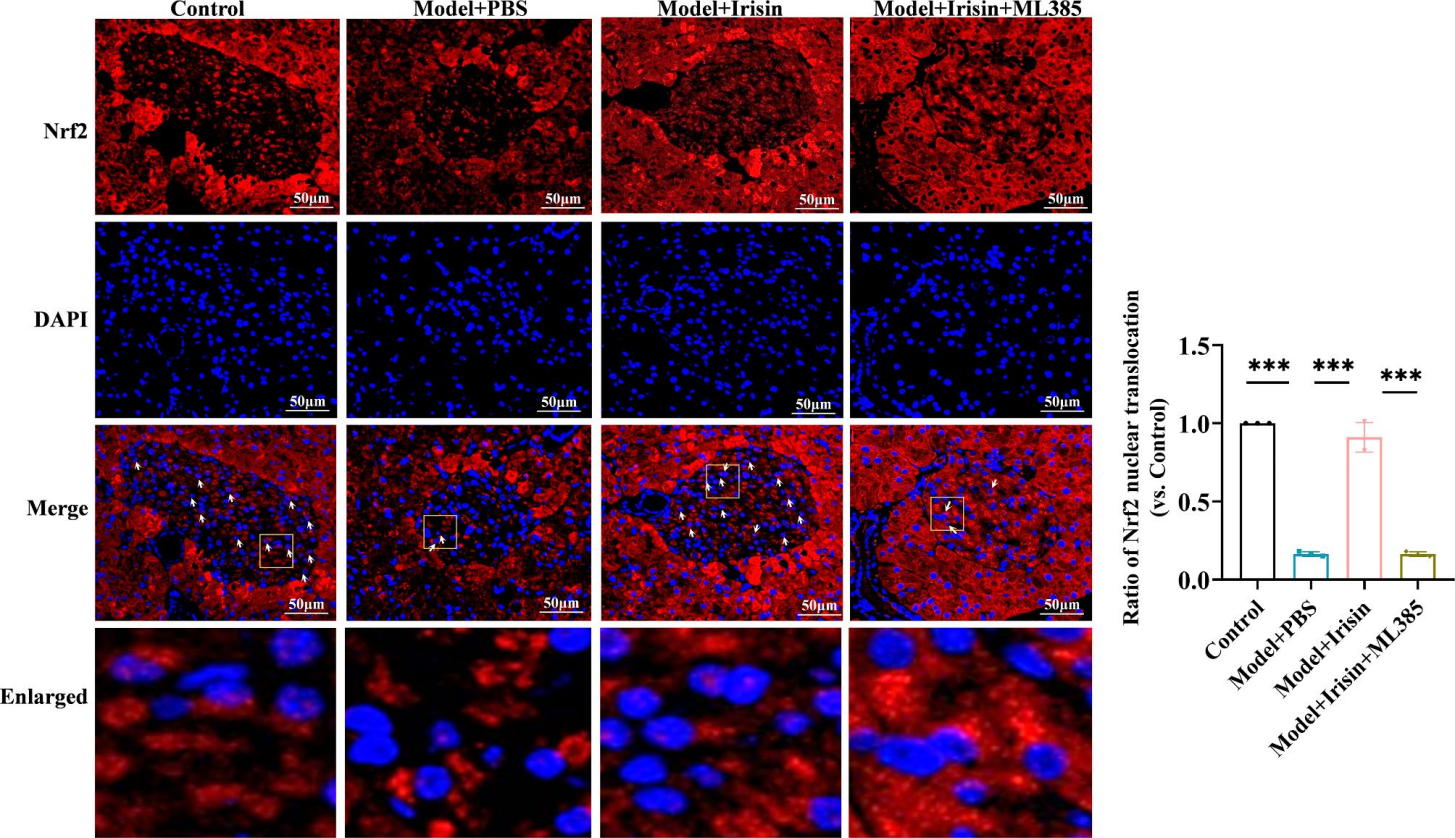
Immunofluorescence of Nrf2 in the nucleus was evaluated; scale bar = 0 μm; ***p < 0.001

**Fig. 7:**
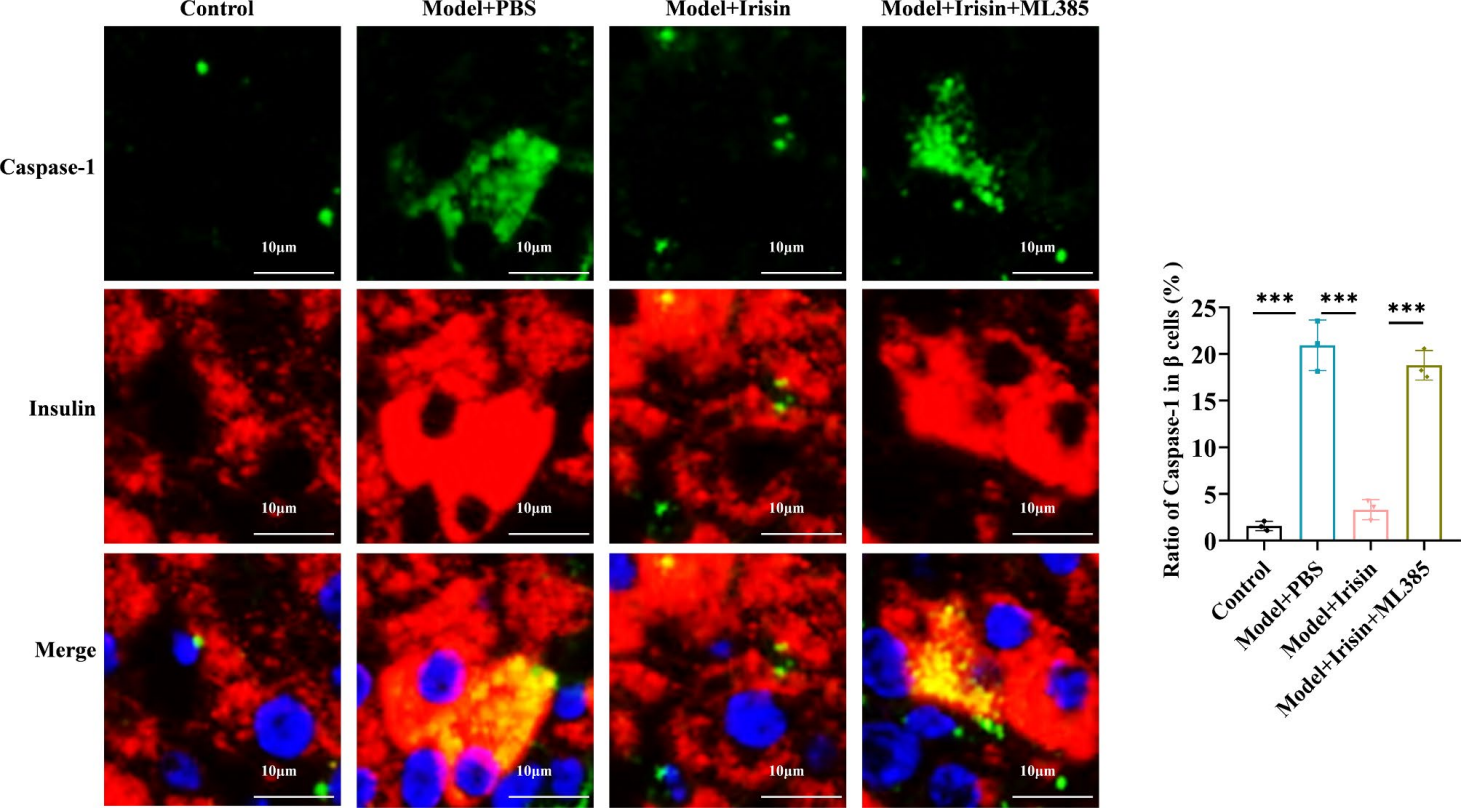
Caspase-1 and β-cell immunofluorescence double-labeling was performed; scale bar = 10 μm; ***p < 0.001

**Fig. 8:**
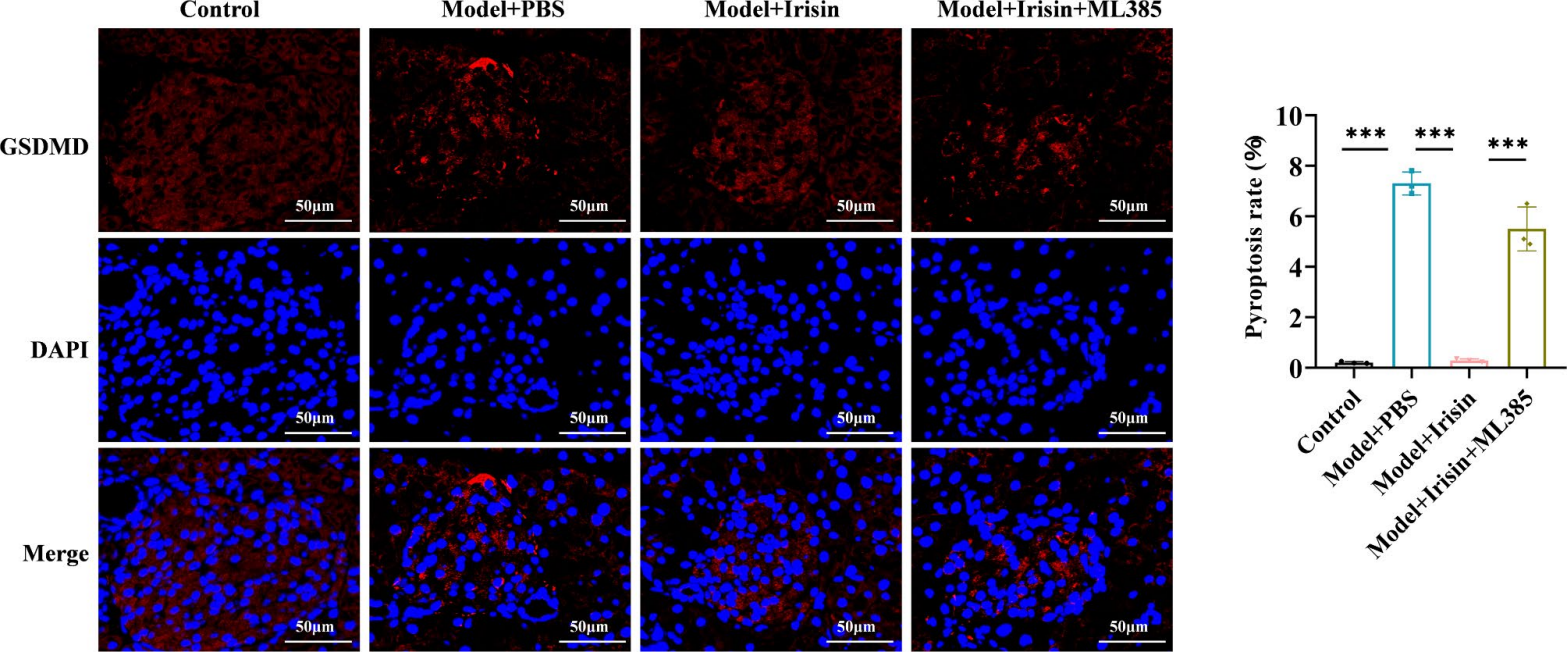
GSDMD immunofluorescence staining was performed; scale bar = 50 μm; ***p < 0.001

Finally, we investigated the mechanism by which irisin inhibits oxidative stress levels and NLRP3 expression by activating the Nrf2-TrX/TXNIP signaling axis in mice. First, we examined ROS production, MDA levels, and SOD activity in the pancreatic tissues of mice. The mice in the diabetic model group showed significantly higher ROS and MDA levels and lower SOD activity. Irisin significantly decreased the release of ROS, reduced the MDA levels, and enhanced SOD activity. These effects of irisin were reversed after treatment with ML385 (Fig. 5A-C). The results of the Western blotting assays showed a considerable reduction in the expression of Nrf2 and TrX1 in the nuclei of pancreatic cells among mice belonging to the diabetic model group. In contrast, the expression of TXNIP, NLRP3, ASC, C-caspase-1, and GSDMD-N increased significantly. Irisin reversed the protein expression level of these markers. After treatment with ML385, the irisin-mediated effects on the changes in the expression of the TXNIP, NLRP3, ASC, C-caspase-1, and GSDMD-N proteins increased significantly, and the expression levels of Nrf2 and TrX1 decreased significantly (Fig. 5D and E). The results of the immunofluorescence staining assays showed that irisin increased the proportion of Nrf2 entering the nucleus, which was counteracted by ML385 (Fig. 6). Irisin significantly decreased caspase-1 levels, but this effect disappeared after treatment with ML385 (Fig. 7). The results of the immunofluorescence staining assays for GSDMD showed similar results (Fig. 8). Thus, the results of our in vitro and in vivo experiments showed that irisin decreased oxidative stress levels and pyroptosis by activating the Nrf2-TrX/TXNIP signaling axis.

## Discussion

The pathogenesis of T2DM includes insulin resistance and defective pancreatic β-cell function; specifically, defective β-cell function plays a key role. Studies have shown that irisin has beneficial effects on metabolic disorders, such as obesity and T2DM [25]. Although the application of irisin is promising, further studies are needed to fully understand its specific mechanism of action. In this study, we investigated the protective effect of irisin on HG-induced pancreatic β-cell pyroptosis. We found that irisin inhibits oxidative stress and cell pyroptosis through the Nrf2-TrX/TXNIP signaling axis. Therefore, we constructed a mouse model to validate this mechanism in vivo. Our findings provided a theoretical foundation for determining novel molecular targets to postpone the failure of β-cell function and effective strategies for treating T2DM.

Pyroptosis significantly contributes to the failure and impaired functioning of β-cells and plays a key role in the progression of T2DM. A chronic inflammatory response occurs in T2DM [26]. The exposure of mouse or human pancreatic islet β-cells to high glucose induces the production of IL-1β, which is toxic to β-cells [27]; also, the production of IL-1β is accompanied by pyroptosis [28]. The secreted IL-1β undergoes a series of signaling processes that promote β-cell pyroptosis and decrease insulin secretion [29]. The inflammatory response of β-cells can lead to β-cell pyroptosis and, thus, mediate the development of pancreatic β-cell defects [30]. In our study, HG-induced Min6 cells in mice secreted high levels of IL-1β and IL-18 and activated GSDMD and caspase-1, which were associated with pyroptosis.

Several studies have reported low serum irisin levels in individuals diagnosed with T2DM. Irisin can impart protection by stimulating the growth of pancreatic β cells and impeding apoptosis [31]. In another study, we found that the inflammatory response initiated by β cells can result in pyroptosis, subsequently causing dysfunction in the islet β cells [24]. Combined treatment with guava (*Psidium guajava*) and alginate was found to improve pancreatic β-cell function by decreasing caspase-3-mediated apoptosis, LC3-B-mediated cellular autophagy, and IL-1β-mediated cellular pyroptosis, which protects the kidneys and pancreas from the HG-induced damage of T2DM [15]. Our findings showed that the expression levels of inflammatory factors and cellular pyroptosis-related molecules were significantly inhibited after treatment with irisin. We confirmed the effect of irisin in vivo using a mouse model of T2DM; our findings showed that irisin significantly restored β-cell function in these mice.

The development of T2DM is significantly influenced by oxidative stress. Oxidative stress activates several signaling pathways, and the activation of these pathways interferes with insulin function. These changes lead to insulin resistance and induction of pancreatic islet β-cell exhaustion and hypoplasia, which in turn contribute to the onset and progression of T2DM [32]. Pyroptosis mediated by activation of the NLRP3 inflammasome aggravated diabetic rat myocardial ischemia/reperfusion injury, whereas silencing the NLRP3 gene ameliorated diabetic cardiomyopathy in T2DM rats [33]. In this study, we found that irisin considerably decreased oxidative stress levels and inhibited the expression of the NLRP3 inflammasome. The activation of Nrf2 antioxidant signaling attenuates the level of oxidative stress in the body and helps maintain redox homeostasis. Dl-NBP attenuates Alzheimer-like lesions by activating the Nrf2-TrX/TXNIP signaling axis, which leads to the inhibition of NLRP3 inflammatory vesicles [34]. Similarly, in cerebral ischemia-reperfusion injury, Nrf2 inhibits NLRP3 inflammatory vesicles by regulating the Trx/TXNIP complex to inhibit the activity of NLRP3 inflammatory vesicles [35]. The findings of these studies suggested that activation of the antioxidant Nrf2-TrX/TXNIP signaling axis plays an important role in reducing oxidative stress and inhibiting NLRP3-mediated pyroptosis. No study has investigated whether irisin regulates the pyroptosis of pancreatic islet β-cells through the Nrf2-TrX/TXNIP signaling axis and NLRP3 inflammasome in a T2DM model. To investigate this mechanism, we used the Nrf2 inhibitor ML385 and assessed the nuclear translocation of Nrf2; we also estimated the levels of TrX/TXNIP, NLRP3 inflammasome, and pyroptosis-related molecules. Our findings indicated that inhibiting Nrf2 in pancreatic β-cells significantly counteracted the antioxidant effects of irisin, as well as, its ability to inhibit NLRP3 inflammasome and the expression of caspase-1, IL-1β, IL-18, and GSDMD-N. We found that irisin can protect islet β cells by inhibiting the expression of NLRP3 through the Nrf2-TrX/TXNIP signaling pathway, decreasing pyroptosis, and reducing the level of IL-1β.

## Conclusion

To summarize, in this study, we investigated the effects of irisin on pyroptosis in Min6 cells and T2DM model mice and elucidated its mechanism of action. Our findings suggested that irisin can decrease HG-induced pyroptosis in islet β-cells in vitro and also in T2DM model mice. We also elucidated its mechanism of action and showed that irisin inhibited pyroptosis and oxidative stress by activating the Nrf2-TrX/TXNIP signaling axis and suppressing the NLRP3-GSDMD pathway. Our findings provided a theoretical foundation for identifying new molecular targets that might delay the failure of islet β-cells and determining better strategies to treat T2DM.

### Electronic supplementary material

Below is the link to the electronic supplementary material.

Supplementary Material 1

Supplementary Material 2

## Data Availability

All data generated or analysed during this study are included in this article.
