# Supplementary material for: Irisin suppresses pancreatic β cell pyroptosis in T2DM by inhibiting the NLRP3-GSDMD pathway and activating the Nrf2-TrX/TXNIP signaling axis
Source: Diabetol Metab Syndr. 2023 Nov 22;15:239. doi: 10.1186/s13098-023-01216-5 (PMC10664367; doi:10.1186/s13098-023-01216-5)
Supplement: Supplementary file 1 — Supplementary Material 1 [file 13098_2023_1216_MOESM1_ESM.docx]

1. Irisin inhibits pyroptosis and oxidative stress by inhibiting NLRP3-GSDMD pathway.

2. Irisin activates the Nrf2-TrX/TXNIP signaling axis in T2DM model cells and mice.

3. Irisin improved islet cell function and insulin resistance in diabetes model mice

4. Irisin has potential therapeutic effects on T2DM.
