# Supplementary material for: Irisin suppresses pancreatic β cell pyroptosis in T2DM by inhibiting the NLRP3-GSDMD pathway and activating the Nrf2-TrX/TXNIP signaling axis
Source: Diabetol Metab Syndr. 2023 Nov 22;15:239. doi: 10.1186/s13098-023-01216-5 (PMC10664367; doi:10.1186/s13098-023-01216-5)
Supplement: Supplementary file 2 — Supplementary Material 2 [file 13098_2023_1216_MOESM2_ESM.ppt]

## Slide 1
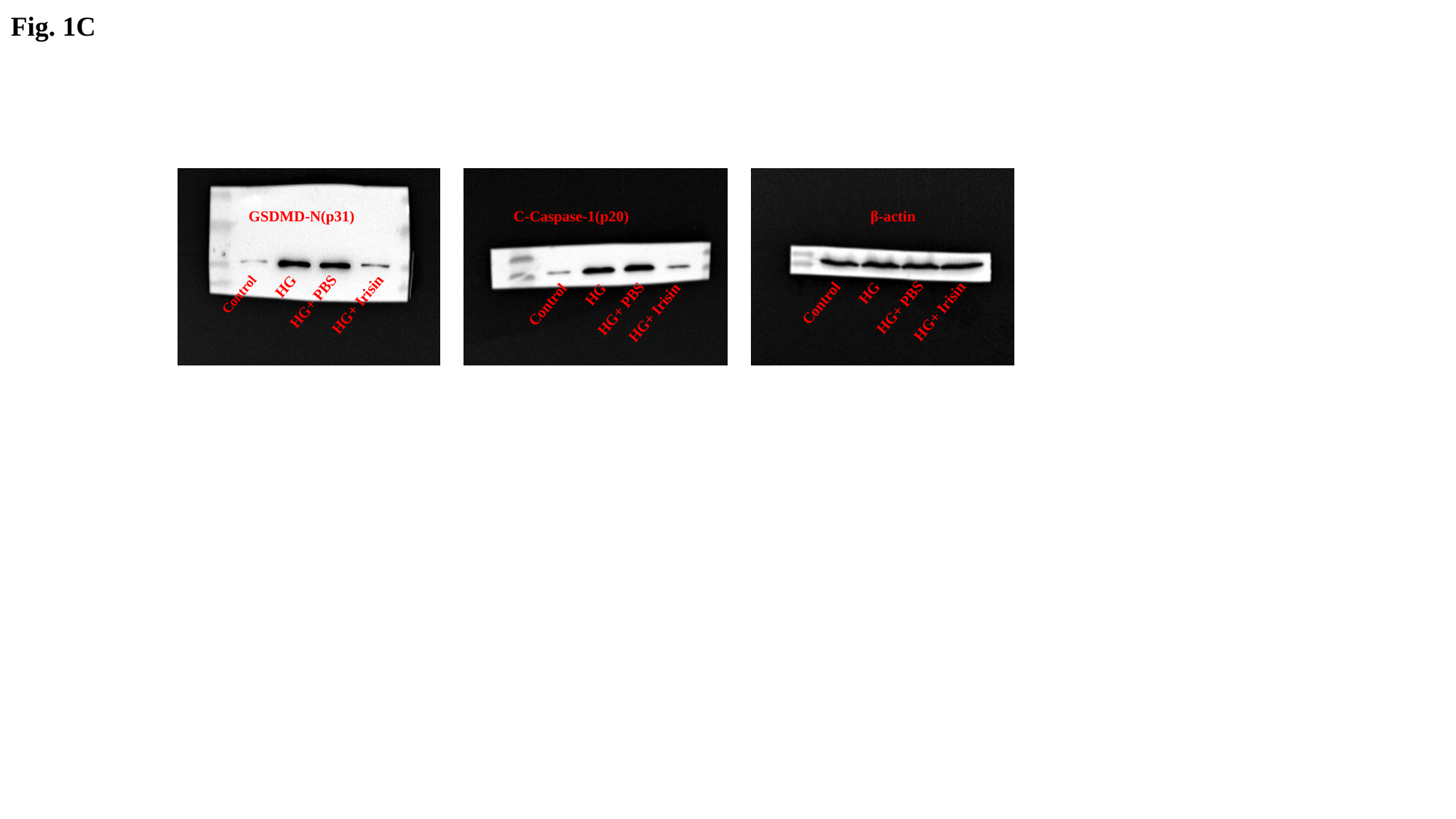

Fig. 1C
GSDMD-N(p31)
C-Caspase-1(p20)
β-actin
Control
HG+ PBS
Control
HG+ Irisin
Control
HG
HG+ PBS
HG+ PBS
HG+ Irisin
HG+ Irisin
HG
HG

## Slide 2
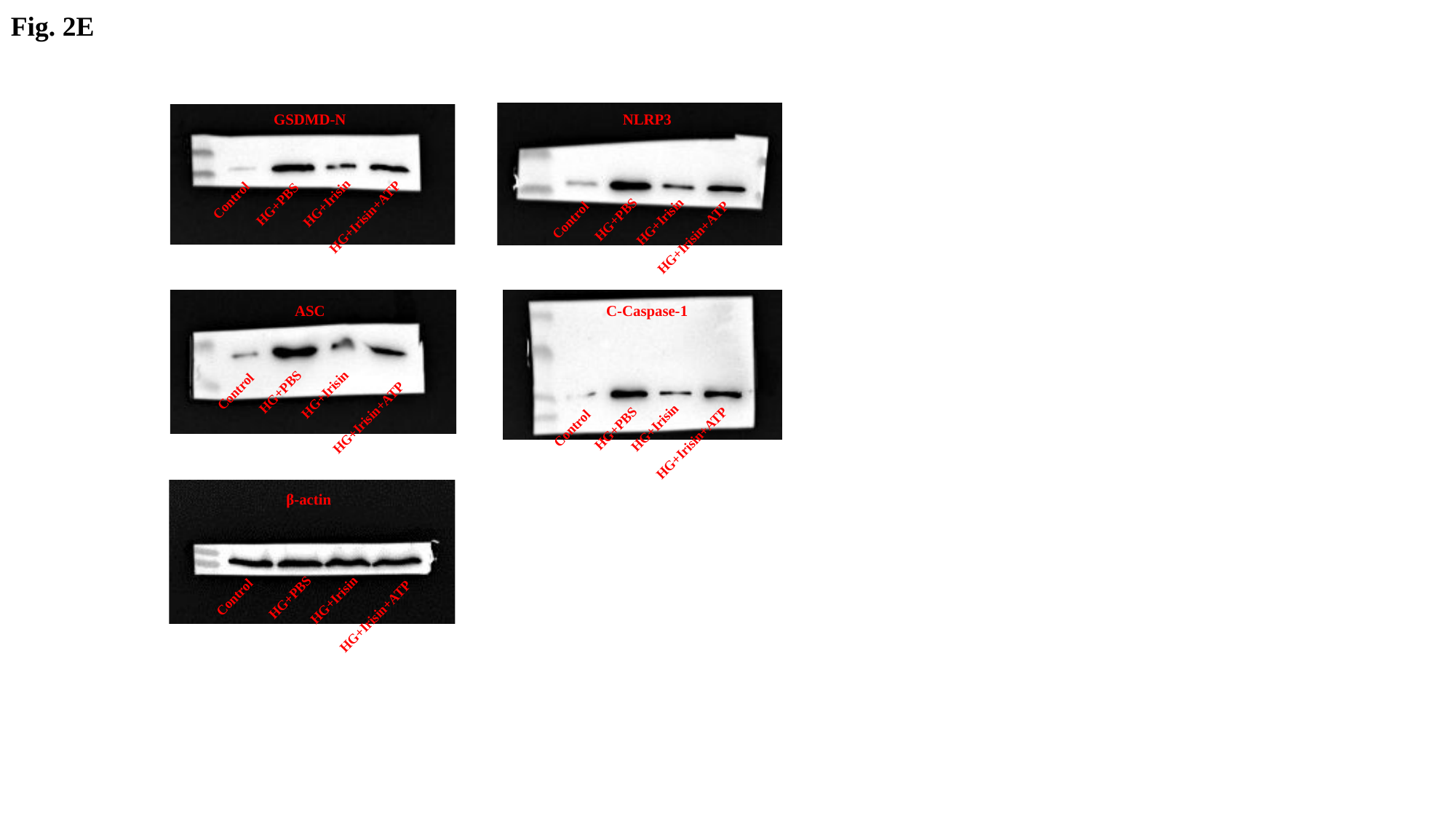

Fig. 2E
NLRP3
Control
HG+PBS
HG+Irisin
GSDMD-N
Control
HG+PBS
HG+Irisin
HG+Irisin+ATP
HG+Irisin+ATP
ASC
Control
HG+PBS
HG+Irisin
C-Caspase-1
Control
HG+PBS
HG+Irisin
HG+Irisin+ATP
HG+Irisin+ATP
β-actin
Control
HG+PBS
HG+Irisin
HG+Irisin+ATP

## Slide 3
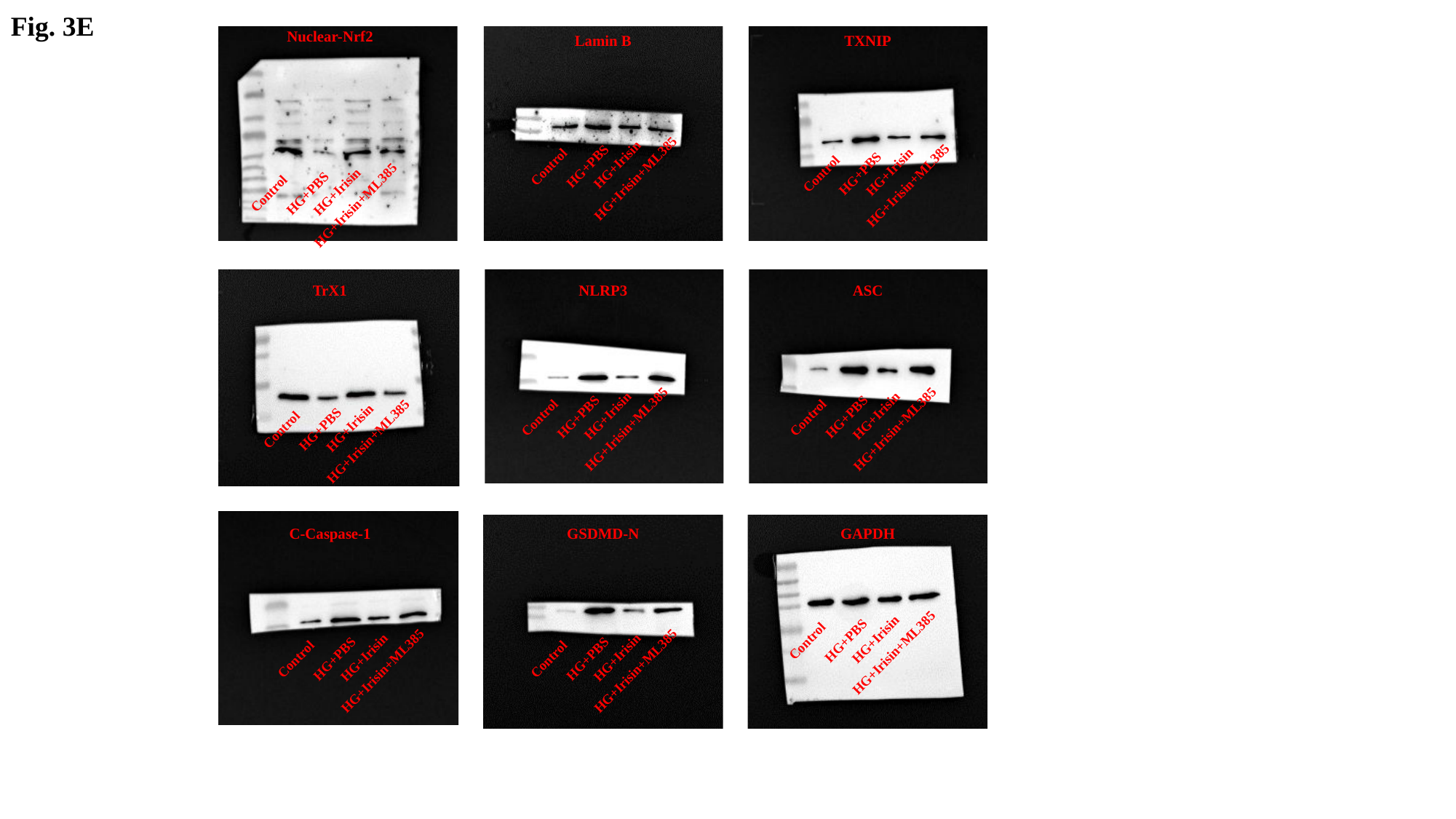

Fig. 3E
Nuclear-Nrf2
Lamin B
TXNIP
Control
HG+PBS
HG+Irisin
Control
HG+PBS
HG+Irisin+ML385
HG+Irisin
HG+Irisin+ML385
Control
HG+PBS
HG+Irisin
HG+Irisin+ML385
TrX1
NLRP3
ASC
Control
Control
HG+PBS
HG+PBS
HG+Irisin
HG+Irisin
HG+Irisin+ML385
HG+Irisin+ML385
Control
HG+PBS
HG+Irisin
HG+Irisin+ML385
C-Caspase-1
GSDMD-N
GAPDH
Control
HG+PBS
HG+Irisin
HG+Irisin+ML385
Control
Control
HG+PBS
HG+PBS
HG+Irisin
HG+Irisin
HG+Irisin+ML385
HG+Irisin+ML385

## Slide 4
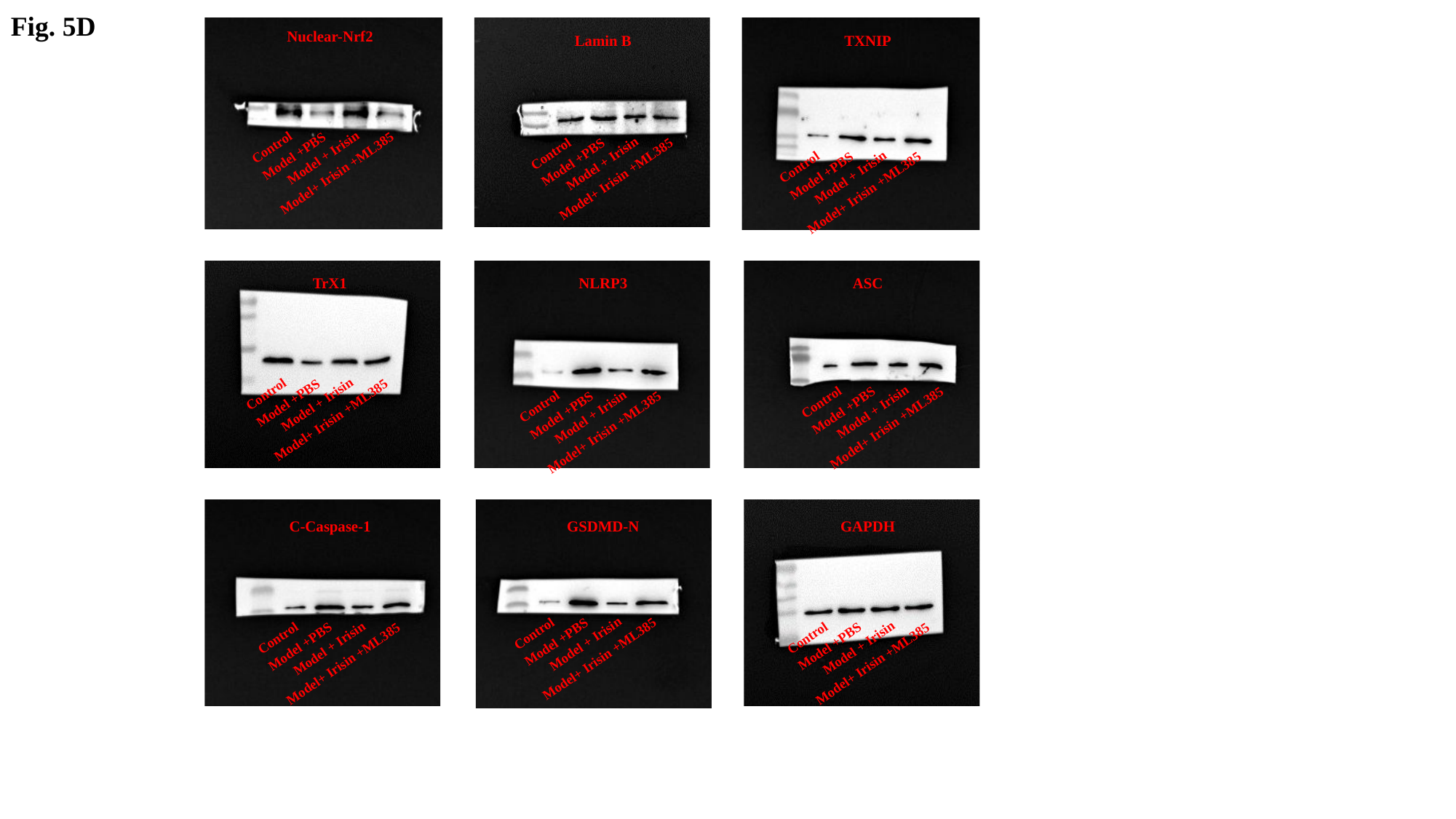

Fig. 5D
Nuclear-Nrf2
Lamin B
TXNIP
Control
Model +PBS
Model + Irisin
Model+ Irisin +ML385
Control
Model +PBS
Model + Irisin
Model+ Irisin +ML385
Control
Model +PBS
Model + Irisin
Model+ Irisin +ML385
TrX1
NLRP3
ASC
Control
Model +PBS
Model + Irisin
Model+ Irisin +ML385
Control
Model +PBS
Model + Irisin
Model+ Irisin +ML385
Control
Model +PBS
Model + Irisin
Model+ Irisin +ML385
C-Caspase-1
GSDMD-N
GAPDH
Control
Model +PBS
Model + Irisin
Model+ Irisin +ML385
Control
Model +PBS
Model + Irisin
Model+ Irisin +ML385
Control
Model +PBS
Model + Irisin
Model+ Irisin +ML385
